# Geometric Reliability of Super-Resolution Reconstructed Images from Clinical Fetal MRI in the Second Trimester

**DOI:** 10.1007/s12021-023-09635-5

**Published:** 2023-06-07

**Authors:** Tommaso Ciceri, Letizia Squarcina, Alessandro Pigoni, Adele Ferro, Florian Montano, Alessandra Bertoldo, Nicola Persico, Simona Boito, Fabio Maria Triulzi, Giorgio Conte, Paolo Brambilla, Denis Peruzzo

**Affiliations:** 1 NeuroImaging Laboratory, Scientific Institute IRCCS Eugenio Medea, Bosisio Parini, Italy; 2 Department of Information Engineering, University of Padua, Padua, Italy; 3 Department of Pathophysiology and Transplantation, University of Milan, Milan, Italy; 4 Social and Affective Neuroscience Group, IMT School for Advanced Studies Lucca, Lucca, Italy; 5 Department of Neurosciences and Mental Health, Fondazione IRCCS Ca’ Granda Ospedale Maggiore Policlinico, Milan, Italy; 6 Department of Woman, Child and Newborn, Fondazione IRCCS Ca’ Granda Ospedale Maggiore Policlinico, Milan, Italy; 7 Department of Services and Preventive Medicine, Fondazione IRCCS Ca’ Granda Ospedale Maggiore Policlinico, Milan, Italy; 8 Padova Neuroscience Center, University of Padua, Padua, Italy

**Keywords:** Fetal brain, Magnetic Resonance Imaging, Super-Resolution Algorithm, Fetal biometry, Pediatric Imaging

## Abstract

Fetal Magnetic Resonance Imaging (MRI) is an important noninvasive diagnostic tool to characterize the central nervous system (CNS) development, significantly contributing to pregnancy management. In clinical practice, fetal MRI of the brain includes the acquisition of fast anatomical sequences over different planes on which several biometric measurements are manually extracted. Recently, modern toolkits use the acquired two-dimensional (2D) images to reconstruct a Super-Resolution (SR) isotropic volume of the brain, enabling three-dimensional (3D) analysis of the fetal CNS.

We analyzed 17 fetal MR exams performed in the second trimester, including orthogonal T2-weighted (T2w) Turbo Spin Echo (TSE) and balanced Fast Field Echo (b-FFE) sequences. For each subject and type of sequence, three distinct high-resolution volumes were reconstructed via NiftyMIC, MIALSRTK, and SVRTK toolkits. Fifteen biometric measurements were assessed both on the acquired 2D images and SR reconstructed volumes, and compared using Passing-Bablok regression, Bland-Altman plot analysis, and statistical tests.

Results indicate that NiftyMIC and MIALSRTK provide reliable SR reconstructed volumes, suitable for biometric assessments. NiftyMIC also improves the operator intraclass correlation coefficient on the quantitative biometric measures with respect to the acquired 2D images. In addition, TSE sequences lead to more robust fetal brain reconstructions against intensity artifacts compared to b-FFE sequences, despite the latter exhibiting more defined anatomical details.

Our findings strengthen the adoption of automatic toolkits for fetal brain reconstructions to perform biometry evaluations of fetal brain development over common clinical MR at an early pregnancy stage.

**Supplementary Information:**

The online version contains supplementary material available at 10.1007/s12021-023-09635-5.

## Introduction

Fetal Magnetic Resonance Imaging (MRI) or in utero MRI is an important noninvasive diagnostic tool in the field of prenatal diagnosis, and its use has widely spread during the last two decades thanks to a combination of advances in imaging and analysis technology, coupled with the high availability of MRI scanners. Although ultrasound remains the first imaging modality in the examination of the fetal central nervous system, some abnormalities cannot be adequately characterized by ultrasound alone (Manganaro et al., 2017). In such cases, MRI may play a crucial role in improving the diagnosis thanks to its superior image resolution and tissue contrast (Griffiths et al., 2017), thus having a significant impact on pregnancy management (Moltoni et al., 2021; Weisstanner et al., 2015).

Prenatal brain MRI routine practice relies on morphologic assessment and biometric measurement evaluation. In clinical practice, fetal brain MRI biometry is an effective indicator of neurodevelopment and is performed on a series of two-dimensional (2D) images acquired via anatomical sequences (e.g., T2-weighted (T2w) Turbo Spin Echo (TSE) or balanced Fast Field Echo (b-FFE) sequences) (Conte et al., 2018). In particular, fast 2D sequences, acquired over different planes and with anisotropic voxels, are recommended with respect to three-dimensional (3D) sequences because of their minor susceptibility to the fetal movement (Glenn et al., 2010).

Biometric measurements are manually extracted in each of the three orthogonal planes (axial, sagittal, and coronal) and then compared to reference values (Conte et al., 2018; Kyriakopoulou et al., 2017). Automated methods for the computation of biometric measurements in a highly complex and rapidly changing brain morphology could improve the diagnostic and decision-making process. However, while several automatic approaches for the computation of ultrasound-based biometric linear measurements are provided (Khan et al., 2017; van den Heuvel et al., 2018; Al-Bander et al., 2019), in MRI only a few algorithms are available, e.g. for the evaluation of the cerebral biparietal diameter, the bone biparietal diameter, and the transcerebellar diameter (Avisdris et al., 2021a, b). These methods mimic the radiologist’s manual annotation workflow, but in some cases lack accuracy in the segmentation of the fetal brain or in the selection of the slice to be used for the measurements.

Novel advanced image processing techniques based on super-resolution (SR) algorithms handle multiple 2D fetal scans, most likely corrupted by motion artifacts, and reconstruct a high-resolution brain volume with an isotropic voxel size. This approach introduces the possibility of evaluating the fetal brain biometry, navigating the reconstructed image over any plane, not only the acquired ones. Moreover, SR reconstructed volumes enable true 3D structures segmentation, which is arduous from conventional 2D slice-wise imaging protocols (Uus et al., 2022). Existing reconstruction frameworks (Rousseau et al., 2006; Jiang et al., 2007; Kim et al., 2010; Gholipour et al., 2010; Kuklisova-Murgasova et al., 2012; Kainz et al., 2015; Alansary et al., 2017; Hou et al., 2018; Ni et al., 2021; Song et al., 2022) generally rely on an iterative approach that operates motion correction and Super-Resolution Reconstruction (SRR) (Ebner et al., 2020). These techniques usually handle only part of the whole processing pipeline (i.e., fetal brain localization, segmentation, robust reconstruction, and template-space alignment) and require a laborious and time-consuming tuning of multiple hyper-parameters. On the other hand, a fully automatic tool addressing all processing steps and validated over different acquisition protocols is highly recommended to achieve efficacious and accurate fetal brain reconstructions. Nowadays, only three modern tools that provide all the functionality for fetal brain reconstruction from MR scans are available: NiftyMIC (Ebner et al., 2020), Medical Image Analysis Laboratory Super-Resolution ToolKit (MIALSRTK) (Tourbier et al., 2015, 2020) and 3D UNet-driven Slice to Volume Reconstruction ToolKit (SVRTK) (Kuklisova-Murgasova et al., 2012).

Previous MRI studies have been conducted to compare qualitatively and/or quantitatively 2D images with 3D SR reconstructions. Kyriakopoulou et al. (2017) and Khawam et al. (2021) conducted biometric assessments on both 2D acquired images and SR reconstructions generated by SVRTK and MIALSRTK, respectively. Their results suggest that biometric measurements extracted from 2D images and 3D reconstructions are highly correlated without significant differences. However, their analyses were performed on a wide gestational age range (18–38 weeks), with very few samples at GA lower than 21 weeks (6 and 2 subjects, for Kyriakopoulou et al., 2017 and Khawam et al., 2021, respectively). Uus et al. (2022) directly compared, for the first time, the reconstructions generated by different SR algorithms (NiftyMIC, MIALSRTK, and SVRTK), mainly focusing on the motion artifacts characterization in the acquired images and their impact on volume reconstructions. The comparison among the different SR algorithms was primarily based on the computational times required to reconstruct the fetal brain, while only a qualitative comparison was carried out on the reconstructed images.

In this study, we characterized qualitatively and quantitatively the geometric reliability of the fetal brain SR reconstruction obtained via the three above-mentioned modern tools (i.e., NiftyMIC, MIALSRTK, and SVRTK). We specifically focused on a narrow gestational age range of 20–21 weeks, which is recognized as a crucial diagnostic period in the course of pregnancy (Prayer et al., 2017). In fact, the early diagnosis of developmental anomalies during this period can have significant implications for pregnancy management (Conte et al., 2018) and may also have legal implications in some countries where legal pregnancy termination is allowed up to a certain gestational age. Despite being a challenging context due to the high level of motion (Uus et al., 2022), these specific GAs are often underrepresented in the datasets and poorly investigated (as in Kyriakopoulou et al., 2017; Khawam et al., 2021). In detail, we assessed the geometric reliability of the brain SR reconstructions by comparing the biometric measures derived from the acquired 2D images with those obtained from the SR reconstructions on a heterogeneous dataset of fetal MRI images. Furthermore, we examined two different acquisition sequences (i.e., TSE and b-FFE) to evaluate which of them led to more reliable measures and high-resolution reconstructions.

## Methods

### Dataset

#### Population

17 fetal brain MR imaging examinations of singleton pregnancies (GAs: 20.24 ± 0.44 weeks) were collected at the Scientific Institute IRCCS Fondazione Ca’ Granda Ospedale Maggiore Policlinico (Milan, Italy).

Exclusion criteria for mothers include (1) twin pregnancy, (2) history of perinatal adverse events, (3) infective or autoimmune diseases, (4) use of systemic corticosteroids, and (5) congenital, genetic, or neurological disorders. Exclusion criteria for the fetus include congenital, genetic disorders and the presence of brain malformation in the acquired MR images.

The procedures were approved by the institutional ethical review boards of the hospital, and all women signed an informed consent for the research use of data.

#### MRI Data

Fetal MR data were acquired with an Achieva d-Stream 3T Philips scanner (Best, The Netherlands) using a phased-array abdominal coil. The fetal brain MR imaging protocol included T2w TSE and/or b-FFE (i.e., balanced gradient echo in Philips scanners) sequences which were acquired with different Fields Of View (FOV), i.e. Reduced (R) or Wide (W), due to the clinical contexts. Some subjects were also acquired with multiple sequence setups and for each given setup at least one sequence was acquired for each orthogonal orientation. Details on the different MR image acquisition parameters and acquired subjects can be found in Table 1.

**Table 1:** MRI acquisition parameters of different types of T2w TSE and b-FFE sequences. The table reports for each sequence the number of exams, GAs in weeks, number of series, in-plane resolution (mm), slice thickness (mm), slice gap (mm), echo time (ms), repetition time (ms). GAs, echo time and repetition time are discussed in terms of minimum-maximum value, mean and standard deviation (SD). The subjects were acquired with multiple sequence setups

Sequences	Number of exams	GAs (weeks)	Number of series	In-plane resolution (mm)	Slice thickness (mm)	Slice gap (mm)	Echo time (ms)		Repetition time (ms)	
		Mean ± SD					Min - Max	Mean ± SD	Min - Max	Mean ± SD
TSE R-FOV	11	20.18 ± 0.40	85	0.44	2.5	1	180	180	3500	3500
TSE W-FOV	12	20.25 ± 0.45	103	0.47	3	3	180	180	3500	3500
b-FFE R-FOV	7	20.29 ± 0.49	50	0.68	3	-	4.5–4.8	4.6 ± 0.1	9.0 - 10.0	9.3 ± 0.2
b-FFE W-FOV	10	20.40 ± 0.52	53	0.71	3	1	4.5–4.8	4.7 ± 0.1	9.0 - 10.0	9.3 ± 0.2

### Super-Resolution Reconstruction

**Fig. 1:**
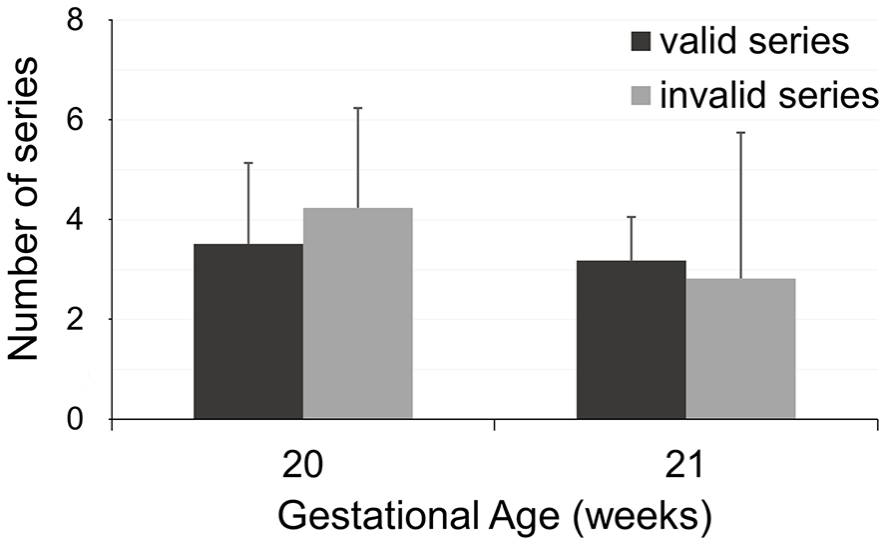
Distribution of the acquired sequences, divided on the basis of the fetus GA (20 and 21 weeks) and the visual inspection results (valid and discarded series). Both series distributions are shown in terms of mean values and standard deviation. Valid series were subsequently used to compute the SR volumes

For each subject, the orthogonal MR sequences of the fetal brain were reconstructed into SR volumes via the publicly available toolkits NiftyMIC^1^ (v0.8), MIALSRTK^2^ (v2.03), SVRTK^3^ (v0.2), following their recommended pipelines. Before the reconstruction, all the images acquired with different sequences and different setups were divided into subsets containing homogeneous images and then were visually inspected to discard sequences with high levels of motion distortion and/or intensity signal dropout (Khawam et al., 2021). On average, 3.35 sequences per subject were used for the reconstruction (Fig. 1). The high rate of discarded images is mainly due to fetal motion, which tends to increase with decreasing fetal age (Uus et al., 2022).

### Qualitative Evaluation of the SR Brain Volumes

**Fig. 2:**
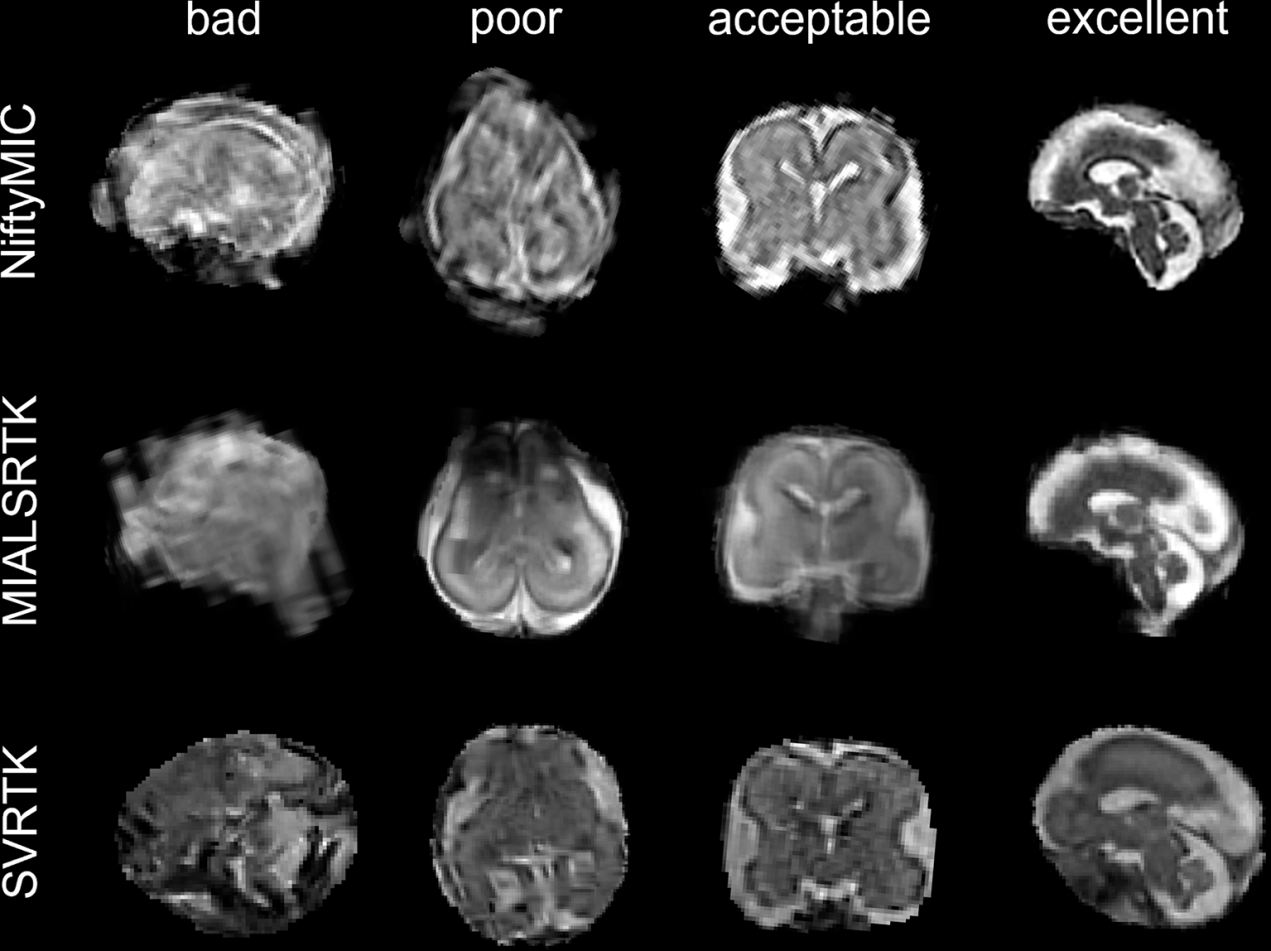
Example of fetal brain Super-Resolution Reconstructed (SRR) quality. The reconstructed brains were rated from bad to excellent

The quality of the brain volume reconstruction was judged in a blinded protocol by two MR pediatric image experts. Reconstructed brain volumes were rated with a Likert scale (Likert et al., 1932) from 1 to 4 (Fig. 2) where a rating of 1 indicates a *bad* quality of fetal brain volume reconstruction, unusable for biometric purposes due to motion distortion and blurring effects; 2 indicate a *poor* quality of fetal brain volume reconstruction, that can be used at least for one reliable biometric measure due to an overall not good quality with still some motion distortion and blurring effects; 3 indicate an *acceptable* quality of fetal brain volume reconstruction, that can be used for biometric purposes due to an overall good quality, but with some blurring effects still relevant; 4 indicate an *excellent* quality of fetal brain volume reconstruction, without any blurring effects.

### Biometric Measurements

The biometric measures were assessed both on the acquired 2D images and SR reconstructions, via the 3D Slicer image computing platform (Fedorov et al., 2012). Biometric measurements were performed in each subject by at least one expert in MR pediatric image analysis. The Intraclass Correlation Coefficient (ICC) was computed on the subjects analyzed by multiple operators to investigate possible dependencies in the acquired measures. The one-way ANOVA statistical test was performed to explore significant differences in the ICCs measures according to the image type (i.e., 2D image and SR reconstructions).

**Fig. 3:**
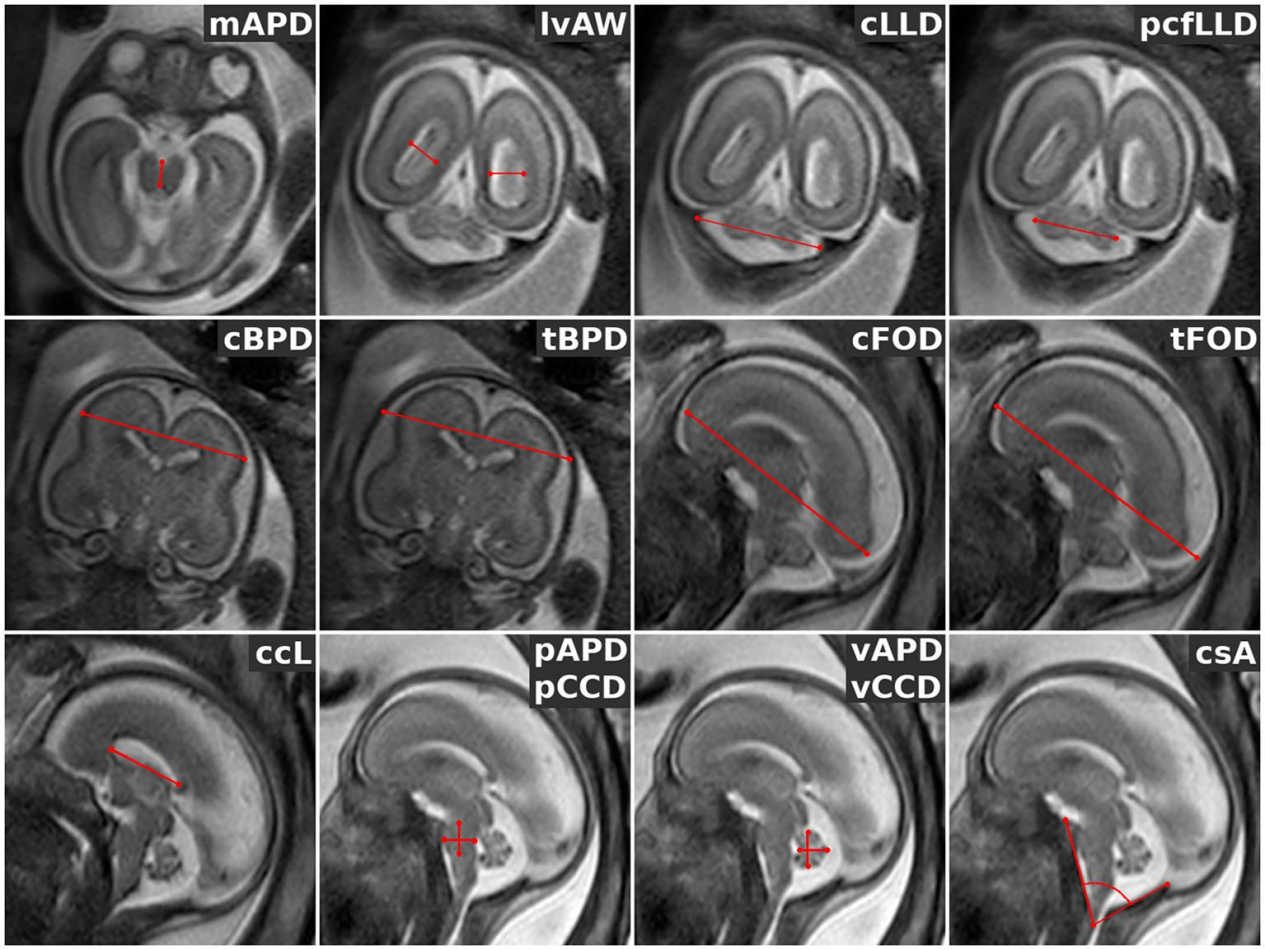
Biometric measurements representation (marked in red) in each orthogonal orientation. For axial orientation, the mesencephalic Antero-Posterior Diameter (mAPD); for coronal orientation the lateral ventricles Atrial Width (lvAW), the cerebellar Latero-Lateral Diameter (cLLD), the posterior cranial fossa Latero-Lateral Diameter (pcfLLD), the cerebral BiParietal Diameter (cBPD), the thecal BiParietal Diameter (tBPD); for sagittal orientation the cerebral Fronto-Occipital Diameter (cFOD), the thecal Fronto-Occipital Diameter (tFOD), the corpus callosum Length (ccL), the pontine Antero-Posterior Diameter (pAPD), the pontine Cranio-Caudal Diameter (pCCD), the vermian Antero-Posterior Diameter (vAPD), the vermian Cranio-Caudal Diameter (vCCD), and the clivo-supraoccipital Angle (csA). For a more detailed description of how to perform the measurements please refer to Conte et al. (2018)

In accordance with the guidelines described in previous studies (Garel et al., 2005; Parazzini et al., 2008; Woitek et al., 2014; Conte et al., 2018) we selected the following biometric measures (Fig. 3): for axial orientation the mesencephalic Antero-Posterior Diameter (mAPD); for coronal orientation the lateral ventricles Atrial Width (lvAW), the cerebellar Latero-Lateral Diameter (cLLD), the posterior cranial fossa Latero-Lateral Diameter (pcfLLD), the cerebral BiParietal Diameter (cBPD), the thecal BiParietal Diameter (tBPD); for sagittal orientation the cerebral Fronto-Occipital Diameter (cFOD), the thecal Fronto-Occipital Diameter (tFOD), the corpus callosum Length (ccL), the pontine Antero-Posterior Diameter (pAPD), the pontine Cranio-Caudal Diameter (pCCD), the vermian Antero-Posterior Diameter (vAPD), the vermian Cranio-Caudal Diameter (vCCD), and the clivo-supraoccipital Angle (csA). All MR imaging measures were expressed in millimeters, with the only exception being the csA in degrees. Each measure was taken two to three times on each acquired 2D image and SR reconstruction, and then averaged on the subject.

### Statistical Analysis

#### Tools Evaluation

An agreement analysis between the biometric measures on each orthogonal 2D acquisition (reference measure) and on the brain volume SR reconstructions (estimated measure) was performed using the Passing-Bablok regression analysis with the Person’s correlation coefficient (Passing & Bablok et al., 1983) and the Bland-Altman plot (Bland & Altman et al., 1999) as in Cardinale et al. (2014). Additionally, the reliability index that reflects both degrees of correlation and agreement between measurements obtained in the SR reconstructions and those obtained in the 2D sequences was evaluated using the ICC, and the criteria outlined by Koo and Li (2016) was adopted to interpret its magnitude. Finally, some related statistical analyses were performed. The Shapiro-Wilk method (Shapiro & Wilk et al., 1965) has been used to test the normality of the distribution of the biometric measures. The mean values and the Standard Deviations (SD) of the biometric measures were compared with a paired two-tailed t-test and F-test.

#### Tools Comparison

A qualitative comparison between the SR reconstructions was performed using the visual inspection scores described above. Moreover, the measurement percentage error between the SR reconstructions and the acquired 2D images was estimated and analyzed using the Passing-Bablok regression. In detail, the inter-rater reliability of the brain volume reconstruction quality categorical assessment was evaluated using Gwet’s agreement coefficient (Gwet’s AC1) and to qualify the magnitude of this coefficient the Altman’s benchmarking was adopted (Gwet et al., 2014). Furthermore, the slope coefficients and the intercepts of the Passing-Bablok regression line were compared with the paired two-tailed t-test and F-test.

#### Sequences Evaluation

The analyses introduced in the previous steps (i.e., image visual inspection, percentage error calculation, Passing-Bablok regression-related test, and further statistical analysis as t- and F-test) were performed splitting the dataset according to the two acquisition sequences (i.e., TSE and b-FFE) to investigate differences in the SR images associated with the acquisition sequence.

All statistical analyses were performed with R software v4.0.5 (R Core Team 2021).

## Results

Forty fetal brain volumes were reconstructed for each SR algorithm (Table 1).

**Fig. 4:**
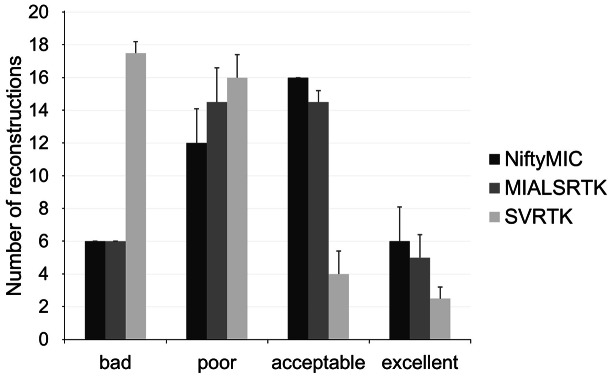
NiftyMIC, MIALSRTK and SVRTK comparison in terms of fetal brain reconstructions quality. Each bar and whisker represent the average and standard deviation consensus among the two raters’ assessments for each quality scale (bad, poor, acceptable, and excellent), respectively

The quality of the reconstructions was rated by two experts, as depicted in Fig. 4. The estimated GWet’s AC1 between the two raters was 0.83. According to Altamn’s benchmarking scale, the magnitude of the estimated coefficient is considered to be *Good* with a probability of 98.8%. For each score value, we considered the quality of reconstruction as the average consensus between the two raters’ assessments. On average, the experts rated 6 NiftyMIC reconstructions, 6 MIALSRTK reconstructions and 17.5 SVRTK reconstructions as *bad*; 12 NiftyMIC reconstructions, 14.5 MIALSRTK reconstructions and 16 SVRTK reconstructions as *poor*; 16 NiftyMIC reconstructions, 14.5 MIALSRTK reconstructions and 4 SVRTK reconstructions as *acceptable*; 6 NiftyMIC reconstructions, 5 MIALSRTK reconstructions and 2.5 SVRTK reconstructions as *excellent.*

Reconstructed volumes scored as *bad* are not usable to derive any quantitative measures for the subsequent analysis. Therefore, 6 (15%), 6 (15%), and 17.5 (44%) volumes were discarded for NiftyMIC, MIALSRTK, and SVRTK, respectively. In addition, due to the large difference in terms of the amount of measures taken between SVRTK and the other methods, we limited the subsequent biometric analyses only to NiftyMIC and MIALSRTK. Thus, 34 fetal brain SR reconstructions obtained via NiftyMIC and MIALSRTK were considered.

### ICC Analysis

In order to investigate the possibility of the measures being influenced by the operator, we calculated the ICC between 3 operators on the measurements performed over 9 fetal brain reconstructions obtained via NiftyMIC and MIALSRTK, and their corresponding 2D images adopted for the reconstruction. The operators’ ICC average between the derived biometric measures on the 2D images was 0.90 with an averaged 95% confidence interval of [0.85–0.94]. According to the criteria outlined by Koo and Li (2016), the operators’ derived measures’ reliability is *Good to Excellent*. The operators’ ICC averaged between the derived measures on the fetal brain SR reconstructions obtained via NiftyMIC was 0.93 with an averaged 95% confidence interval of [0.81–0.98], and via MIALSRTK was 0.88 with an averaged 95% confidence interval of [0.70–0.97]. According to the criteria outlined by Koo and Li (2016), the operators-derived measures’ reliability on NiftyMIC is *Good to Excellent* and on MIALSRTK reconstructions is *Moderate* to *Excellent*, demonstrating the high reliability of the measures among operators. The detailed ICC results obtained for each biometric measure are reported in Table 2.

**Table 2:** Intraclass Correlation Coefficient (ICC) of the biometric measurements performed by three different operators on the 2D images and SR reconstructions

Orthogonal Orientation	Biometric Measure	Operator ICC		
		2D	NiftyMIC	MIALSRTK
AX	mAPD	0.82	0.94	0.76
COR	r-lvAW	0.83	0.91	0.90
	l-lvAW	0.89	0.95	0.94
	cLLD	0.85	0.92	0.91
	pcfLLD	0.95	0.98	0.95
	cBPD	0.95	0.95	0.94
	tBPD	0.94	0.94	0.89
SAG	cFOD	0.91	0.91	0.92
	tFOD	0.84	0.91	0.88
	ccL	0.90	0.94	0.95
	pAPD	0.89	0.95	0.82
	pCCD	0.96	0.92	0.91
	vAPD	0.89	0.96	0.65
	vCCD	0.89	0.84	0.76
	csA	0.95	0.96	0.97

We performed a one-way ANOVA test to investigate whether the image source has an impact on the operator ICC. Statistical results showed a significant dependency of the operator ICC upon the different images (p = 0.027). In particular, the post-hoc analysis indicated that the operator ICC on NiftyMIC reconstructions is significantly larger than those on the MIALSRTK ones (p = 0.025) and 2D images (p = 0.014), while no difference was observed between MIALSRTK reconstructions and 2D images.

### Tools Evaluation

We compared the biometric measures derived from each acquired 2D image (reference method) with those derived from the brain SR reconstruction. For this analysis, we combined all the measures obtained from each acquisition sequence, i.e. independently from the acquired sequence and setup.

It was not possible to estimate all the biometric measurements on each subject subset (i.e., the acquired 2D images or the SR reconstructions) due to a significant number of motion-corrupted low-quality slices both in 2D images and in SR reconstructions. We evaluated 78% of all possible measurements on the 2D images, 65% and 50% of the measurements on the SR brain volumes reconstructed via NiftyMIC and MIALSRTK, respectively (Supplemental Fig. S1).

Measurement means and their SDs are reported for each sequence subset in Table 3. All the measures performed in 2D images and SR reconstructions were normally distributed (p > 0.05). The statistical comparisons between the measurements performed on SR reconstructions and 2D images identified a significant difference in the mean of the cLLD measures for NiftyMIC and MIASLRTK reconstructions (p = 0.01 and p < 0.001 for NiftyMIC and MIALSRTK, respectively). No other significant differences were found for the other mean and SD values.

**Table 3:** Biometric measurements derived from 2D image and reconstructed fetal brain. All the measurements are expressed in millimeters (mm), with the only exception for csA in degrees (°). Each biometric measurement is discussed in terms of mean and standard deviation (SD).

Orthogonal Orientation	Biometric Measure	2D	SRR							
			NiftyMIC				MIALSRTK			
			TSE W-FOV	TSE R-FOV	b-FFE W-FOV	b-FFE R-FOV	TSE W-FOV	TSE R-FOV	b-FFE W-FOV	b-FFE R-FOV
		Mean ± SD	Mean ± SD	Mean ± SD	Mean ± SD	Mean ± SD	Mean ± SD	Mean ± SD	Mean ± SD	Mean ± SD
AX	mAPD (mm)	4.5 ± 0.4	4.5 ± 0.3	4.7 ± 0.3	4.4 ± 0.3	4.5 ± 0.6	4.6 ± 0.5	4.5 ± 0.4	4.5 ± 0.4	4.4 ± 0.3
COR	r-lvAW (mm)	6.2 ± 0.8	7 ± 0.9	6.2 ± 0.9	6.1 ± 0.7	5.6 ± 0.7	6.5 ± 0.9	6.1 ± 0.8	6.2 ± 0.7	5.9 ± 1
	l-lvAW (mm)	6.3 ± 0.7	6.7 ± 0.7	5.9 ± 0.7	6.3 ± 0.9	6.3 ± 0.5	6.3 ± 0.9	6.3 ± 0.4	6.4 ± 0.9	6.5 ± 0.8
	cLLD (mm)	19.6 ± 0.9	19 ± 0.9	19.5 ± 0.8	19.6 ± 1.1	19.3 ± 0.9	18.7 ± 1.3	19.1 ± 1.3	19.4 ± 1.4	19.3 ± 1
	pcfLLD (mm)	27 ± 1.9	27.1 ± 2	27 ± 2.1	26.9 ± 2.1	25.4 ± 1.3	26.4 ± 2.1	27.4 ± 2.9	27.1 ± 2	26.5 ± 1.9
	cBPD (mm)	37.5 ± 1.8	37.5 ± 2.9	37.8 ± 1.9	37.3 ± 2	38.8 ± 2.7	37.3 ± 1.7	36.6 ± 1.9	37.4 ± 2	37.5 ± 2
	tBPD (mm)	44 ± 2.6	44.6 ± 4.1	44.2 ± 2.6	44 ± 2.7	45.7 ± 2.7	44.6 ± 2.7	42.9 ± 2.4	44.4 ± 1.8	44.4 ± 3.5
SAG	cFOD (mm)	49.8 ± 2.5	49.8 ± 2	48.6 ± 2.9	48.8 ± 3	50.2 ± 2.8	50 ± 3.3	48.3 ± 4.5	49.9 ± 2.4	48.2 ± 2.5
	tFOD (mm)	56.2 ± 2.5	56.6 ± 2.1	55.6 ± 2.9	56.4 ± 2.2	56.8 ± 3.1	56.6 ± 2.6	53.3 ± 4.9	57.5 ± 2	55.3 ± 2.2
	ccL (mm)	15.9 ± 1.9	15.5 ± 1.9	15.5 ± 1.9	15.5 ± 1.8	15.2 ± 2.7	14.7 ± 1.8	15.7 ± 2.8	15.1 ± 1.5	14.8 ± 1.6
	pAPD (mm)	6.3 ± 0.5	6.3 ± 0.7	6.4 ± 0.3	6.4 ± 0.5	5.7 ± 0.1	6.3 ± 0.3	6.4 ± 0.8	6.3 ± 0.6	6 ± 0.8
	pCCD (mm)	6 ± 0.6	6.2 ± 0.3	5.9 ± 0.4	6.2 ± 0.7	6.1 ± 0.4	6 ± 0.2	6.1 ± 0.5	6.1 ± 0.6	6.4 ± 0.5
	vAPD (mm)	5.4 ± 0.5	5.3 ± 0.4	5.5 ± 0.6	5.3 ± 0.4	5.5 ± 0.4	5.3 ± 0.6	5.6 ± 0.4	5.5 ± 0.5	5.8 ± 0.4
	vCCD (mm)	7.3 ± 0.7	7 ± 0.9	8.1 ± 0.6	7.1 ± 0.9	7.2 ± 1.3	6.6 ± 0.8	7.3 ± 0.9	7.1 ± 1.1	7.4 ± 0.8
	csA (°)	71.7 ± 7.7	73.4 ± 8.8	70 ± 6.3	71.3 ± 5.8	74.6 ± 5.4	69.2 ± 2.6	73.2 ± 11.3	71.9 ± 8	68 ± 10.8

**Fig. 5:**
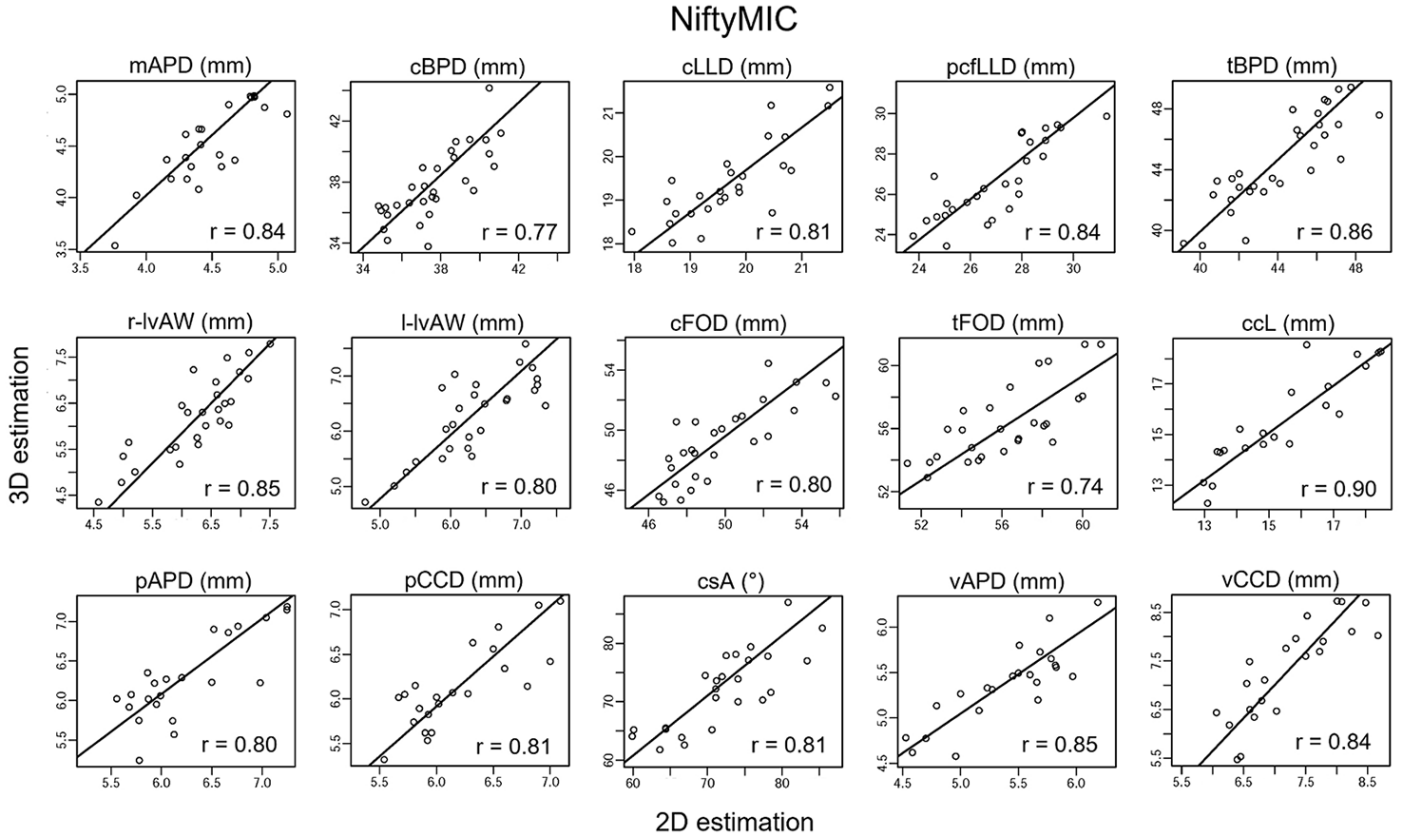
2D and NiftyMIC SR derived biometric measurements estimation agreement. The scatter plots with Passing-Bablok regression lines are presented for each biometric measurement. Each scatter plot shows a significant agreement between 2D and SR reconstruction estimations with the Person’s correlation coefficient (p < 0.003, Bonferroni corrected). The reconstructed fetal brain is obtained via NiftyMIC.

**Fig. 6:**
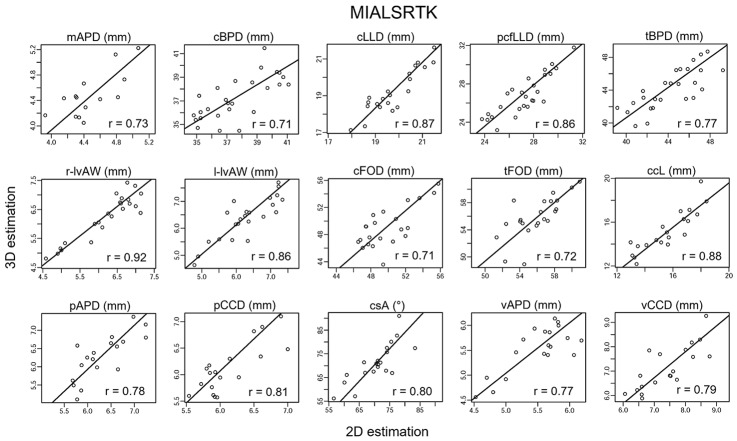
2D and MIALSRTK SR derived biometric measurements estimation agreement. The scatter plots with Passing-Bablok regression lines are presented for each biometric measurement. Each scatter plot shows a significant agreement between 2D image estimations and SR brain reconstruction estimations with the Person’s correlation coefficient (p < 0.003, Bonferroni corrected). The reconstructed fetal brain is obtained via MIALSRTK

Figures 5-6 depict the scatter plots comparing the 2D and SR-derived estimations of the biometric measurements, along with the Passing-Bablok regression lines. All biometric measurements show a significant correlation coefficient (all p < 0.003, Bonferroni corrected) between the estimates derived from the acquired 2D images and those derived from the SR reconstructions. The slope and the intercept values (with a 95% confidence interval) of the Passing-Bablok regression line are reported in Supplemental Table S1.

The Bland-Altman plots of biometric measurements performed on 2D images and tools SR reconstructions confirm the results obtained with the Passing-Bablok test (Supplemental Fig. S2-S3 and Supplemental Table S2).

Finally, the average ICC between the biometric measures was 0.82 with an averaged 95% confidence interval of [0.62–0.92] for NiftyMIC, and 0.79 with a 95% confidence interval of [0.57–0.90] for MIALSRTK. According to the criteria outlined by Koo and Li (2016), the reliability of both tools is *Moderate* to *Good*. The ICC results are reported for each biometric measurement in Table 4.

**Table 4:** Intraclass Correlation Coefficient (ICC) between biometric measurements derived from reconstructed fetal brains and 2D images

Orthogonal Orientation	Biometric Measure	2D-SRR ICC	
		NiftyMIC	MIALSRTK
AX	mAPD	0.85	0.86
COR	r-lvAW	0.87	0.88
	l-lvAW	0.87	0.84
	cLLD	0.77	0.80
	pcfLLD	0.84	0.85
	cBPD	0.74	0.57
	tBPD	0.84	0.78
SAG	cFOD	0.75	0.70
	tFOD	0.73	0.74
	ccL	0.92	0.90
	pAPD	0.64	0.50
	pCCD	0.82	0.91
	vAPD	0.87	0.89
	vCCD	0.88	0.79
	csA	0.87	0.79

### Tools Comparison

From the visual inspection and scoring of the reconstructed images, the estimated GWet’s AC1 between the two raters was 0.74 and 0.78 for NiftyMIC and MIALSRTK, respectively. According to Altamn’s benchmarking scale, the magnitude of the estimated coefficient is considered to be *Good* with a probability of 95.1% and 99.2% for NiftyMIC and MIALSRTK, respectively.

We computed for each toolkit the percentage error (mean ± SD) of the biometric measurements performed on the SR reconstructions with respect to those derived from 2D images (Table 5). Results showed an overall average error rate of -0.1% ± 4.9% and − 0.7% ± 5.1% for NiftyMIC and MIALSRTK, respectively. In 11 out of 15 measurements, NiftyMIC shows a smaller magnitude of the mean percentage error with respect to MIALSRTK, and in 9 out of 15 measurements, it is characterized by a smaller SD.

**Table 5:** Toolkits (NiftyMIC and MIALSRTK) comparison in terms of biometric measurements percentage error. The error is calculated between the measurements derived from the SR reconstructions and those derived from the 2D images. The percentage values are discussed in terms of mean and standard deviation (SD).

Orthogonal Orientation	Biometric Measure	SRR	
		NiftyMIC	MIALSRTK
		Mean ± SD	Mean ± SD
AX	mAPD	0.53% ± 4.64%	0.12% ± 5.51%
COR	r-lvAW	-0.74% ± 7.51%	0.85% ± 4.76%
	l-lvAW	-0.9% ± 7.05%	1.36% ± 6.71%
	cLLD	-1.51% ± 2.86%	-3.31% ± 3.05%
	pcfLLD	-1.32% ± 4.08%	-1.53% ± 4.08%
	cBPD	0.4% ± 3.92%	-0.95% ± 3.92%
	tBPD	0.91% ± 3.45%	-0.01% ± 3.95%
SAG	cFOD	-1.12% ± 3.28%	-1.46% ± 4.38%
	tFOD	0.4% ± 3.22%	-0.08% ± 3.92%
	ccL	0.98% ± 5.45%	-2.46% ± 5.7%
	pAPD	0.5% ± 5.63%	-0.54% ± 6.36%
	pCCD	-0.95% ± 4.59%	-0.16% ± 4.45%
	vAPD	-0.36% ± 4.65%	0.66% ± 5.51%
	vCCD	1.18% ± 7.74%	-3.53% ± 7.66%
	csA	0.2% ± 5.62%	0.68% ± 6.88%

Furthermore, we compared the two toolkits on the Passing-Bablok regression estimates that are reported in Supplemental Table S1. No significant differences were found comparing the toolkits slope and intercept values with the paired two-tailed t-test. Finally, significant differences were found comparing the toolkits intercept values with the F-test (p = 0.02).

### Sequences Evaluation

We investigated which MRI sequence (i.e., TSE or b-FFE) led to more reliable SR brain reconstructions.

From the visual quality assessment of the reconstructions, the estimated GWet’s AC1 between the two raters was 0.89 and 0.64 for TSE and b-FFE reconstructions achieved via NiftyMIC, respectively; and 0.77 and 0.78 for TSE and b-FFE reconstructions achieved via MIALSRTK, respectively. According to Altamn’s benchmarking scale, the estimated coefficient was *Very Good* with a probability of 99.9% for TSE and *Moderate* with a probability of 98.9% for b-FFE reconstructions obtained via NiftyMIC. The estimated coefficient was *Good* with a probability of 93.6% and 93% for both TSE and b-FFE reconstructions obtained via MIALSRTK, respectively.

**Fig. 7:**
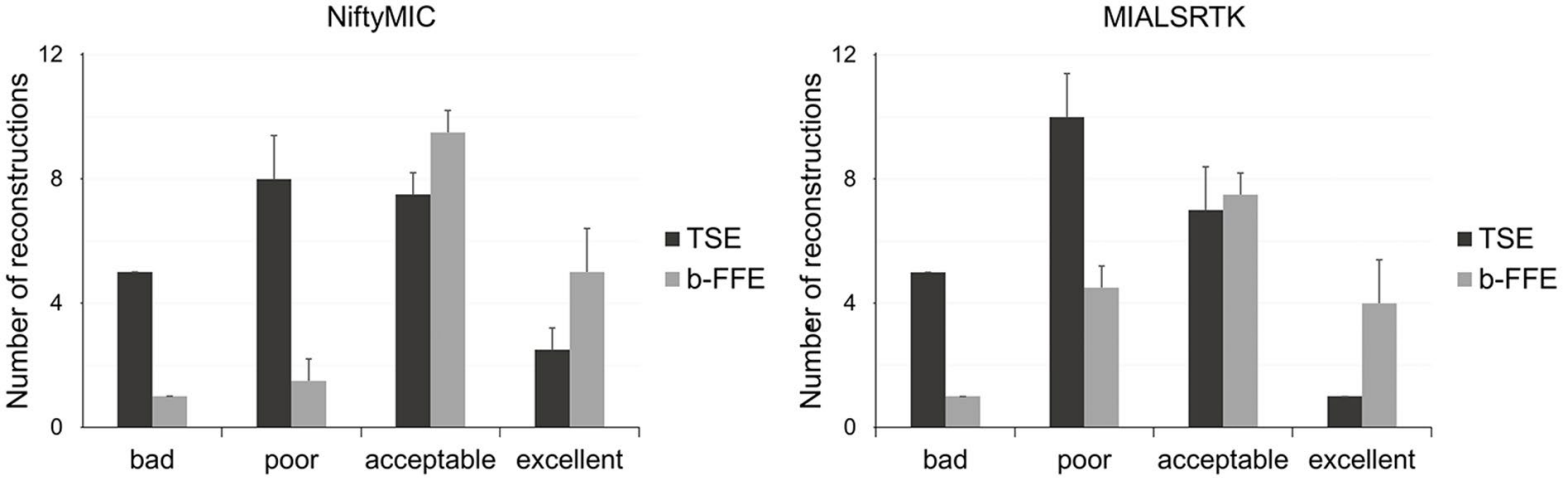
MRI sequences (T2w- TSE and b-FFE) quality comparison of the fetal brain reconstructions obtained via NiftyMIC and MIALSRTK. Each bar and whisker report the quality average and standard deviation consensus among the two raters’ assessments for each quality scale (bad, poor, acceptable, and excellent), respectively

For each score value, we considered the quality of reconstruction as the average consensus between the two raters’ assessments. On average, the experts rated 5 TSE and 1 b-FFE reconstructions via NiftyMIC and MIALSRTK as *bad*; 8 TSE and 1.5 b-FFE reconstructions via NiftyMIC and 10 TSE and 4.5 b-FFE reconstructions via MIALSRTK as *poor*; 7.5 TSE and 9.5 b-FFE reconstructions via NiftyMIC and 7 TSE and 7.5 b-FFE reconstructions via MIALSRTK as *acceptable*; and 2.5 TSE and 5 b-FFE reconstructions via NiftyMIC and 1 TSE and 4 b-FFE reconstructions via MIALSRTK as *excellent* (Fig. 7).

**Fig. 8:**
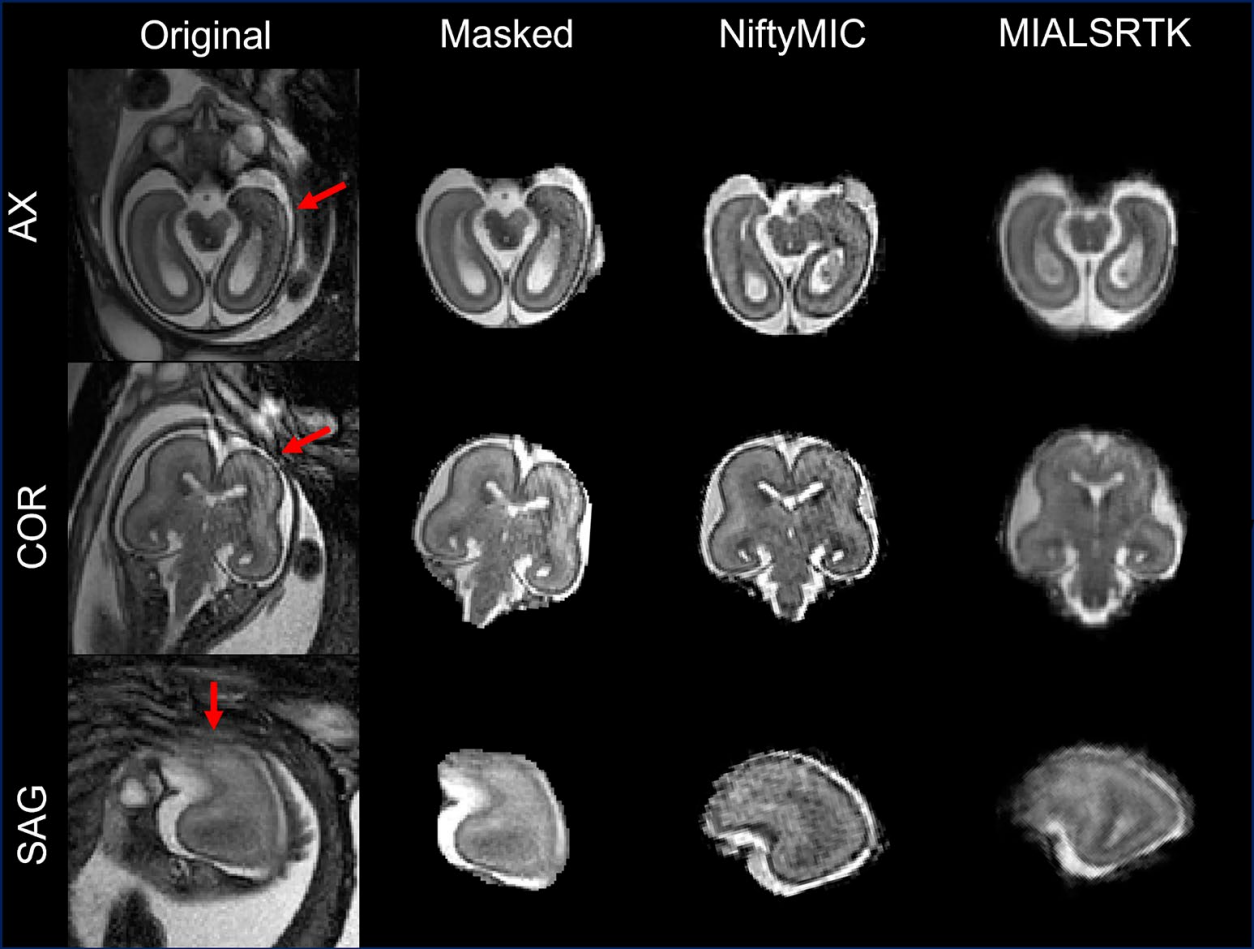
Examples of b-FFE artifacts. The artifact is reported on 2D images (original and its brain mask) and SR reconstructions obtained via NiftyMIC and MIALSRTK. The intensity artifact, pointed out by the red arrow, is shown in each of the three orthogonal planes (axial, sagittal, and coronal)

The visual inspection pointed out that b-FFE sequences were usually characterized by the presence of intensity artifacts due to their susceptibility to field inhomogeneities (Gholipour et al., 2014) affecting both the acquired 2D images and the SR reconstructions, independently from the adopted reconstruction toolkit (Fig. 8).

We estimated the percentage error of the biometric measurements derived from the SR reconstructions from the different sequences with respect to the 2D image-derived ones (Table 6). Results showed an average of the orthogonal orientations error rate of 0.3% ± 4.9% and − 0.4% ± 4.8 for TSE and b-FFE reconstructions via NiftyMIC, respectively; and − 0.75% ± 5.37% and − 0.61% ± 4.8% for TSE and b-FFE reconstructions via MIALSRTK, respectively. The statistical analysis showed that the percentage error of the different measurements was significantly different from 0 only for the ccL measure (p = 0.03) in the b-FFE reconstructions, and vCCD measure (p = 0.03) in the TSE reconstructions via NiftyMIC; and for cLLD measure in b-FFE (p = 0.01) and in TSE (p = 0.004) reconstructions, and for vCCD measure (p = 0.044) in TSE reconstructions via MIALSRTK.

**Table 6:** MRI sequences (T2w TSE and b-FFE) comparison in terms of biometric measurements percentage error. The error is calculated between the measurements derived from the SR reconstructions and those derived from 2D images. The fetal brain reconstructions are obtained via both NiftyMIC and MIALSRTK. The percentage values are discussed in terms of mean and standard deviation (SD)

Orthogonal Orientation	Biometric Measure	NiftyMIC		MIALSRTK	
		TSE	b-FFE	TSE	b-FFE
		Mean ± SD	Mean ± SD	Mean ± SD	Mean ± SD
AX	mAPD	1.42% ± 5.21%	-0.08% ± 4.32%	0.65% ± 5.66%	-0.17% ± 5.76%
COR	r-lvAW	2.84% ± 7.26%	-3.6% ± 6.61%	1.14% ± 6.36%	0.57% ± 2.88%
	l-lvAW	-2.16% ± 6.33%	0.11% ± 7.64%	0.05% ± 7.36%	2.56% ± 6.12%
	cLLD	-1.96% ± 3.06%	-1.12% ± 2.72%	-3.95% ± 3.29%	-2.77% ± 2.86%
	pcfLLD	-1.43% ± 3.75%	-1.24% ± 4.45%	-2.25% ± 4.1%	-0.82% ± 4.09%
	cBPD	-0.34% ± 4.2%	1.04% ± 3.66%	-1.61% ± 4.57%	-0.4% ± 3.34%
	tBPD	0.4% ± 4.04%	1.35% ± 2.89%	-0.73% ± 4.03%	0.6% ± 3.93%
SAG	cFOD	-1.42% ± 3.41%	-0.79% ± 3.23%	-2% ± 4.52%	-1.01% ± 4.4%
	tFOD	-0.17% ± 3.3%	1% ± 3.15%	-1.5% ± 4.03%	1.1% ± 3.57%
	ccL	1.54% ± 5.32%	0.56% ± 5.74%	-1.27% ± 6.14%	-3.66% ± 5.28%
	pAPD	0.82% ± 6.24%	0.21% ± 5.31%	1.16% ± 8.23%	-1.91% ± 4.36%
	pCCD	-0.03% ± 5.13%	-1.72% ± 4.14%	1.56% ± 4.11%	-1.54% ± 4.43%
	vAPD	-0.49% ± 4.97%	-0.24% ± 4.58%	-0.6% ± 4.46%	1.79% ± 6.32%
	vCCD	4.43% ± 5.55%	-1.52% ± 8.46%	-5.19% ± 6.5%	-2.04% ± 8.63%
	csA	0.93% ± 6.05%	-0.6% ± 5.26%	3.31% ± 7.24%	-1.46% ± 6.06%

Furthermore, we compared the two sequences on the Passing-Bablok regression estimates presented in Supplemental Table S3-S4. The one sample t-test applied on the TSE and b-FFE Passing-Bablok regression slope coefficient and intercept values showed significant differences with respect to a null distribution only for the slope coefficient (p = 0.023) of the TSE reconstructions obtained via MIALSRTK. No significant differences were found comparing the sequences Passing-Bablok regression slope coefficient and intercept values with the paired two-tailed t-test. Finally, the F-test showed significant differences between TSE and b-FFE reconstructions obtained via NiftyMIC only for the intercept values (p = 0.03).

## Discussion

Automatic brain reconstruction methods from 2D fetal MR fast scans are crucial to perform quantitative volumetric studies of brain development (Uus et al., 2022). The publicly available toolkits that provide all the functionality for fetal brain reconstruction from 2D MR images are NiftyMIC, MIALSTRK, and SVRTK. These toolkits were proposed and validated on T2w spin echo sequences, and the geometric reliability of the reconstructed images was not evaluated on heterogeneous datasets (i.e., different acquisition setups and MRI sequences). Moreover, they were optimized over a wide range of GAs, ranging from 20 to 37 weeks, but not specifically tested on the early part of this GAs window (as in Kyriakopoulou et al., 2017; Khawam et al., 2021; Uus et al., 2022). In this study, we successfully addressed these points. We first validated the aforementioned methods, then we conducted a qualitative and quantitative comparison among them over a heterogeneous dataset including different acquisition sequences (i.e., T2w TSE and b-FFE) and setups, focusing on early GAs. We showed that NiftyMIC and MIALSTRK provide reliable SR volumes even in this specific context.

In 2022, Uus and colleagues qualitatively investigated the fetal brain reconstructions generated via SVRTK, NiftyMIC, and MIALSRTK on a wide fetal MRI dataset ranging from 20 to 38 weeks. The similar quality of the obtained reconstructions suggested that the choice of the reconstruction toolbox is mainly driven by personal preferences towards a specific method and reconstruction time limit. In particular, SVRTK provided the smallest reconstruction computational times thanks to its multi-parallel C + + implementation. Conversely, on our dataset acquired in the 20th and 21st gestational week, we found that SVRTK provides unreliable reconstructions in 44% of the cases, i.e. images in which biometric measures cannot be taken. Thus, we limited the further analysis to the NiftyMIC and MIALSRTK reconstructions. The percentage error of the biometric measurements performed on the SR reconstructions obtained with NiftyMIC and MIALSRTK is very small (Table 5) with respect to the one derived from 2D images and it is comparable with the measured population range (Table 3). Also, the 2D-SR reconstruction ICC results (Table 4), averaged between the biometric measures, report high scores for NiftyMIC and MIALSRTK, suggesting that reconstructed volumes are geometrically reliable. The quality assessment demonstrated that 85% of reconstructed volumes, obtained via NiftyMIC and MIALSRTK, could be useful for biometric measurements. NiftyMIC reconstructions were qualitatively rated higher than MIALSRTK reconstructions since MIALSRTK brain reconstructions were more blurred and less anatomically defined. This is reflected in the amount of measurements that could be derived from the SR volumes, higher for the NiftyMIC ones with respect to the MIALSRTK ones. Nevertheless, the measurements are very similar between the two methods, as well as their errors, suggesting a good agreement between them.

In addition, the inter-operator ICC results (Table 2) indicate a significant improvement in the level of agreement among the operators when using NiftyMIC reconstructed images, as opposed to the originally acquired 2D images. We ascribe this result to the higher spatial in-plane resolution in each direction (i.e., the small slice thickness) and higher SNR due to the contribution of the multiple acquired sequences. The normative range of the biometric measures is usually small - especially at early GAs - thus even small errors may cause a significant shift in the corresponding fetal growth centile, eventually leading to misdiagnosis and misguided pregnancy management (Warrander et al., 2020). The improvement of the inter-operator ICC is therefore an important achievement supporting the use of SR images even in clinical practice.

Only the cLLD measure was found to be significantly different between measurements obtained from reconstructed volumes and 2D images. Both NiftyMIC and MIALSRTK usually provide larger cLLD values than the corresponding 2D images. This may be due to the larger partial volume affecting the acquired 2D images with respect to the SR reconstructions. The cerebellum shape rapidly changes over the coronal plane and the 2D coronal images may not catch the largest section due to the wide slice thickness (~ 3 mm in our data).

We also evaluated the reliability/robustness of NiftyMIC and MIALSRTK employing two different sequence types (TSE and b-FFE). To the best of our knowledge, this is the first time that SR algorithms were tested and validated on b-FFE images. In detail, in both sequences, the mean percentage error of the measurements performed on the SR reconstructions is very small (Table 6), indicating that both tools provide geometrically reliable reconstructions even starting from a sequence they were not developed for. From a qualitative point of view, reconstructed volumes obtained via NiftyMIC and MIALSRTK from b-FFE sequences were rated, by the two experts, higher than reconstructions obtained from TSE sequences. This is due to the fact that b-FFE sequence reconstructions show more defined anatomical details, because of their higher spatial in-plane resolution (Table 1). However, inspecting the two different types of T2w sequences, we detected some intensity artifacts affecting both the acquired b-FFE 2D images and the derived SR reconstructions (Fig. 8). The presence of intensity artifacts may be an important source of errors for any operation performed on those images, such as image segmentation, parcellation, volume measurements, suggesting that TSE sequences may be more reliable for subsequent volumetric studies performed on the SR reconstructions.

In this method-comparison study, there were some limitations. First of all, the number of acquired subjects as well as the amount of sequences per orthogonal orientation adopted for the reconstruction were limited. Ideally, to ensure a reliable reconstruction, the required number of sequences is determined by the square of the magnification factor of the resolution targeted (Lin et al., 2004; Rousseau et al., 2010) and therefore, increasing the number of stacks per orientation can further increase the reconstruction quality. Secondly, we considered only a narrow range of gestational age, thus comparing the different toolkits in a very specific context. MRI images acquired around the 21st gestational week suffer from a high level of motion, thus stressing the ability of the different tools to account for large movements and to identify corrupted slices. According to Uus et al. (2022), motion correction algorithms implemented in the tested tools fail when facing large rotations (> 60°). Therefore, our conclusion may not necessarily be generalizable to other gestational periods. Nevertheless, the data evaluated in this study represent a standard clinical scenario, and we showed that the SR toolkits could represent a useful tool for the quantitative evaluation of brain development. Lastly, the toolkits were used with the default settings. However, we still obtained reliable SR reconstructions with NiftyMIC and MIALSRTK, and it is reasonable to assume that toolkit parameter optimization will improve the quality of the reconstructions (Payette et al., 2021).

## Conclusion

This study demonstrates the reliability and robustness of NiftyMIC and MIALSRTK applied to common clinical MRI fetal scans. Currently, in clinical practice, only linear biometric measurements derived from 2D images are used to characterize fetal neurodevelopment. We showed that these measurements could also be derived from the SR reconstructions, and we speculated that their evaluation could be more accurate on SR images than on 2D ones. Moreover, the availability of SR reconstructed images with an isotropic voxel size enables the retrieval of three-dimensional features (e.g., volumetric or surface-based), which may provide a more accurate characterization of brain development. Finally, we disclosed that T2w TSE sequences should be recommended for this aim as they are less affected by intensity artifacts that may impact further quantitative analysis.

### Electronic Supplementary Material

Below is the link to the electronic supplementary material.

Supplementary Material 1

## Data Availability

Owing to ethics and privacy limitations, the data will be made available by request which includes a formal project outline and an agreement of data sharing. Further information should be directed and will be fulfilled by the lead contact, Paolo Brambilla [paolo.brambilla1 (at) unimi (dot) it].

## Abbreviations

b-FFE: balance Fast Field Echo
cBPD: cerebral BiParietal Diameter
ccL: corpus callosum Length
cFOD: cerebral Fronto-Occipital Diameter
cLLD: cerebellar Latero-Lateral Diameter
csA: clivo-supraoccipital Angle
FOV: Field Of View
GA: Gestational Age
ICC: Intraclass Correlation Coefficient
lvAW: lateral ventricles Atrial Width
mAPD: mesencephalic Antero-Posterior Diameter
MRI: Magnetic Resonance Imaging
pAPD: pontine Antero-Posterior Diameter
pCCD: pontine Cranio-Caudal Diameter
pcfLLD: posterior cranial fossa Latero-Lateral Diameter
SR: Super-Resolution
SRR: Super-Resolution Reconstruction
T2w: T2-weighted
tBPD: thecal BiParietal Diameter
tFOD: thecal Fronto-Occipital Diameter
TSE: Turbo Spin Echo
vAPD: vermian Antero-Posterior Diameter
vCCD: vermian Cranio-Caudal Diameter

## Footnotes

1 https://github.com/gift-surg/NiftyMIC.

2 https://github.com/Medical-Image-Analysis-Laboratory/mialsuperresolutiontoolkit.

3 https://github.com/SVRTK/svrtk-docker-gpu.
