# Correction to: Geometric Reliability of Super-Resolution Reconstructed Images from Clinical Fetal MRI in the Second Trimester

**DOI:** 10.1007/s12021-023-09642-6

**Published:** 2023-09-19

**Authors:** Tommaso Ciceri, Letizia Squarcina, Alessandro Pigoni, Adele Ferro, Florian Montano, Alessandra Bertoldo, Nicola Persico, Simona Boito, Fabio Maria Triulzi, Giorgio Conte, Paolo Brambilla, Denis Peruzzo

**Affiliations:** 1 NeuroImaging Laboratory, Scientific Institute IRCCS Eugenio Medea, Bosisio Parini, Italy; 2 Department of Information Engineering, University of Padua, Padua, Italy; 3 Department of Pathophysiology and Transplantation, University of Milan, Milan, Italy; 4 Social and Affective Neuroscience Group, IMT School for Advanced Studies Lucca, Lucca, Italy; 5 Department of Neurosciences and Mental Health, Fondazione IRCCS Ca’ Granda Ospedale Maggiore Policlinico, Milan, Italy; 6 Department of Woman, Child and Newborn, Fondazione IRCCS Ca’ Granda Ospedale Maggiore Policlinico, Milan, Italy; 7 Department of Services and Preventive Medicine, Fondazione IRCCS Ca’ Granda Ospedale Maggiore Policlinico, Milan, Italy

**Correction to: Neuroinformatics (2023) 21:549–563**

10.1007/s12021-023-09635-5.

The original version of this article unfortunately contained mistakes.

An incorrect Fig. 4 was used in the proof. The correct image of Fig. 4 is shown below.

**Fig. 4:**
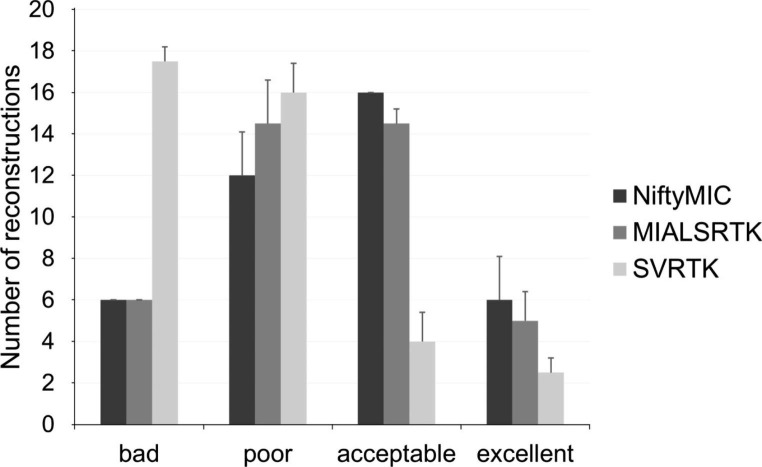
NiftyMIC, MIALSRTK and SVRTK comparison in terms of fetal brain reconstructions quality. Each bar and whisker represent the average and standard deviation consensus among the two raters’ assessments for each quality scale (bad, poor, acceptable, and excellent), respectively

The original article has been corrected.

