# Supplementary material for: Geometric Reliability of Super-Resolution Reconstructed Images from Clinical Fetal MRI in the Second Trimester
Source: Neuroinformatics. 2023 Jun 7;21(3):549–63. doi: 10.1007/s12021-023-09635-5 (PMC10406722; doi:10.1007/s12021-023-09635-5)
Supplement: Supplementary file 1 — Supplementary Material 1 [file 12021_2023_9635_MOESM1_ESM.docx]

**SUPPLEMENTARY MATERIAL**


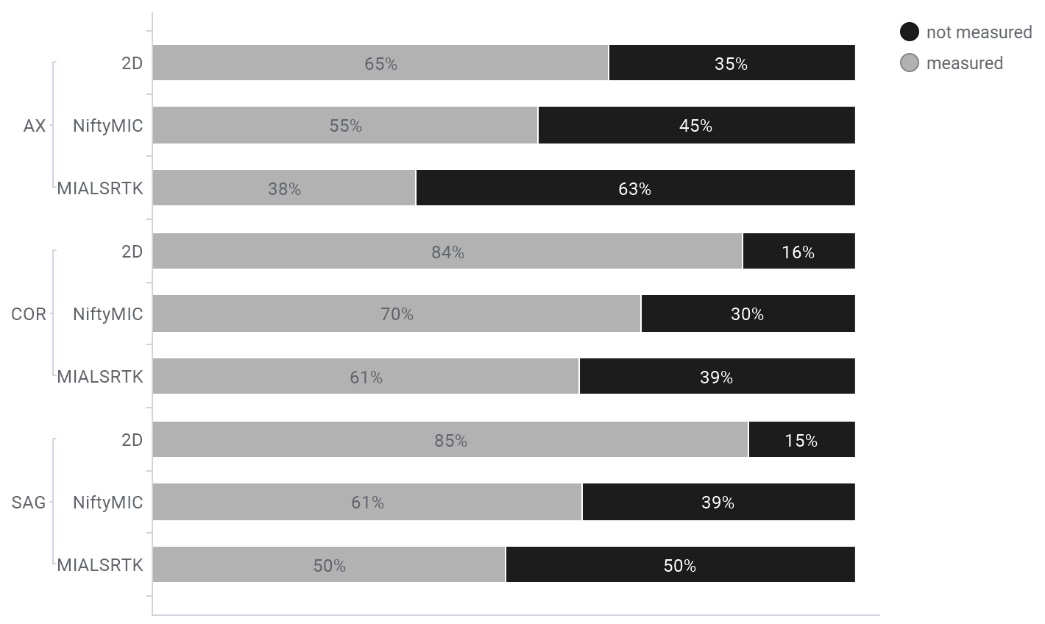


**Supplemental Fig. S1** Performed measurements out of the total for each 2D acquisition and SR reconstruction. The measurements were performed in each orthogonal orientation (axial, sagittal, and coronal) of the different 2D images and SR reconstructions obtained via NiftyMIC and MIALSRTK.


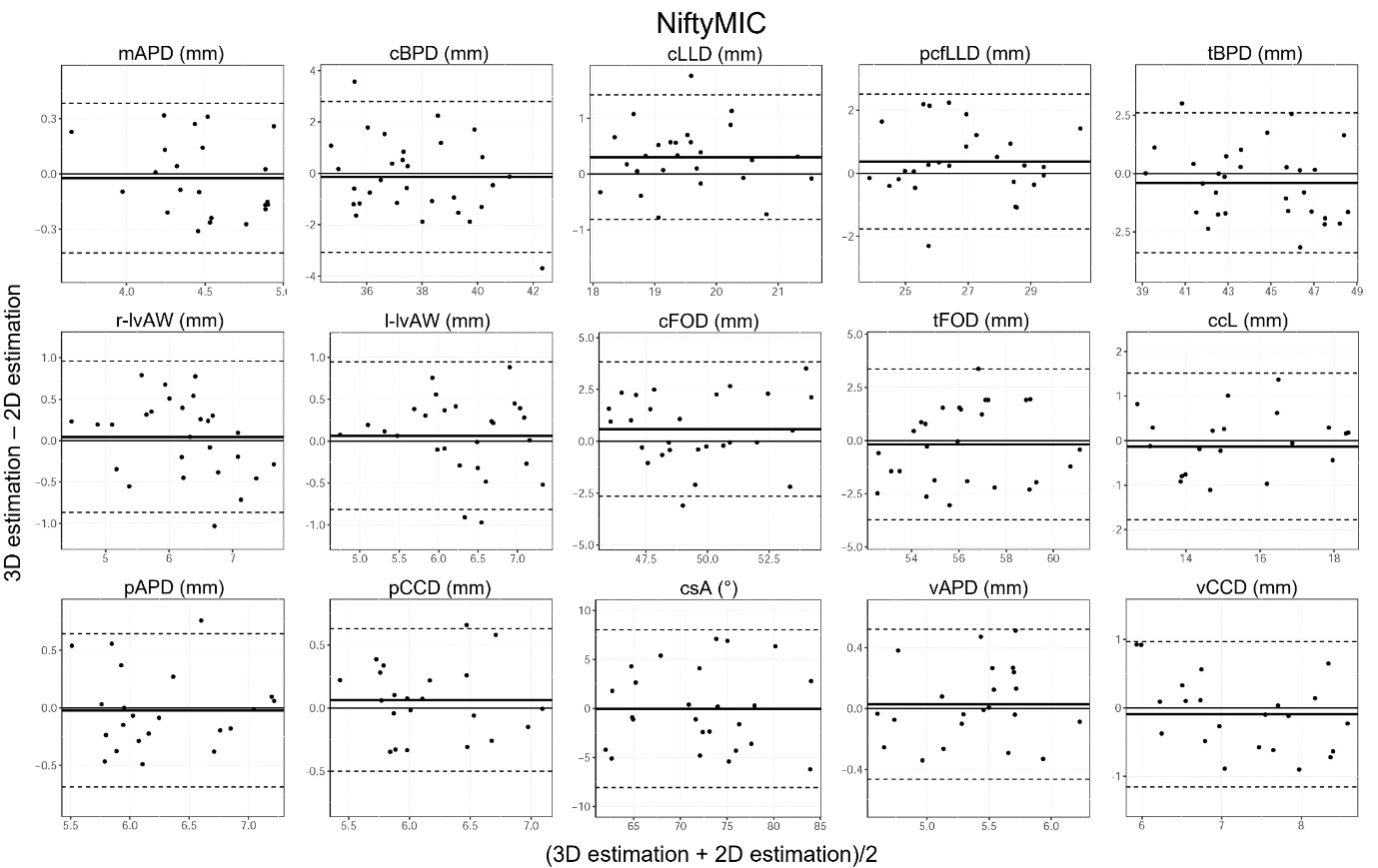


**Supplemental Fig. S2** Bland-Altman plots for 2D and NiftyMIC reconstruction estimations for the biometric measurements. In each plot the mean of all measurement differences (bold black line) is around the perfect identity line (gray line) and included in the 95 % Confidence Interval (dashed lines), suggesting a good agreement between the two measurements.


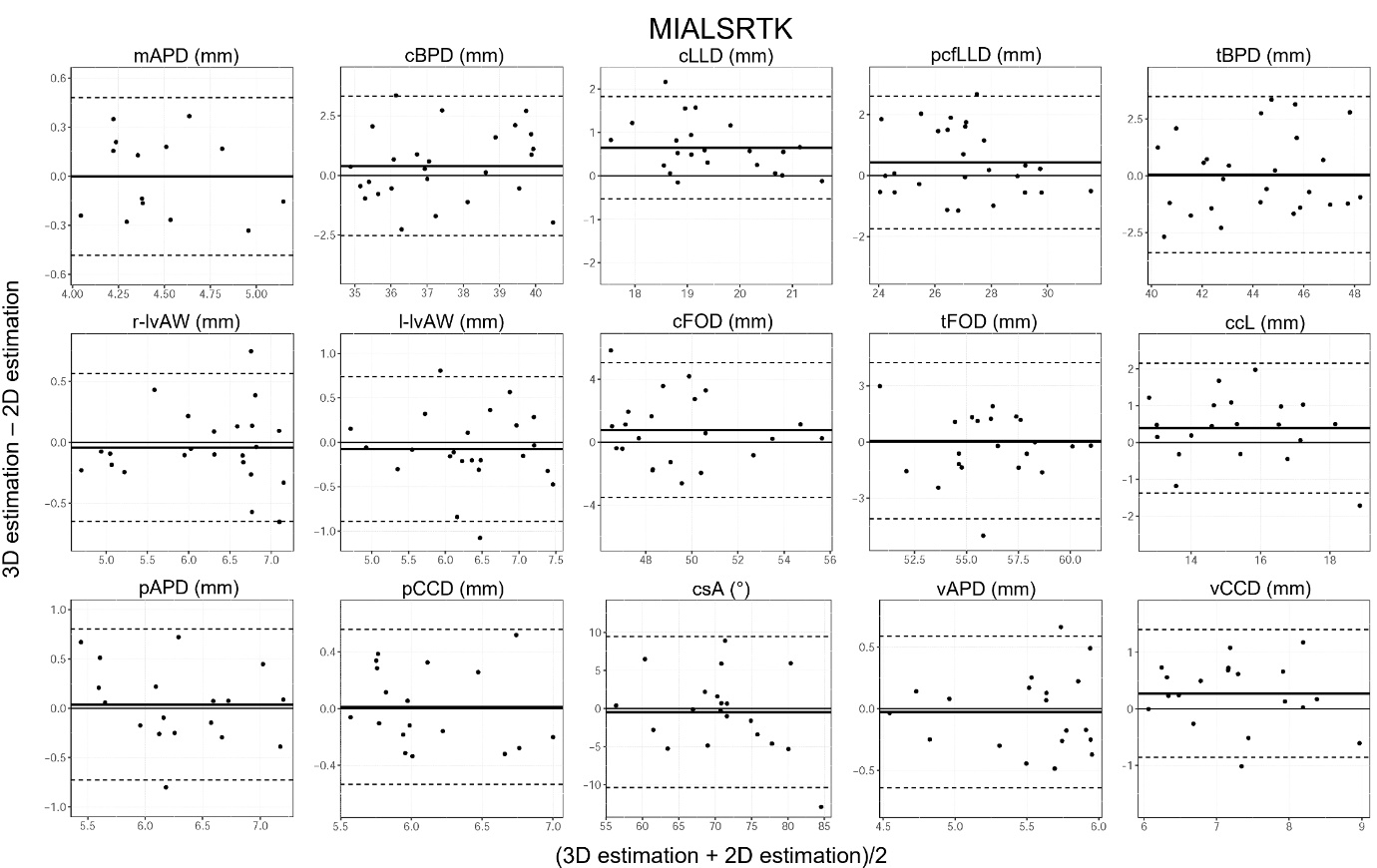


**Supplemental Fig. S3** Bland-Altman plots for 2D and MIALSRTK reconstruction estimations of biometric measurements. In each plot the mean of all measurement differences (bold black line) is around the perfect identity line (gray line) and included in the 95 % Confidence Interval (dashed lines), suggesting a good agreement between the two measurements.

**Supplemental Table S1** NiftyMIC and MIALSRTK comparison in terms of slope and intercept values, derived from the biometric measurements. The slope (m) and intercept (q) values, reported with the 95% Confidence Interval (CI), are estimated using the Passing-Bablok regression analysis on each biometric measurement.

| **Orthogonal**  **Orientation** | **Biometric Measure** | **NiftyMIC** | | **MIALSRTK** | |
| --- | --- | --- | --- | --- | --- |
|  |  | **m (CI)** | **q (CI)** | **m (CI)** | **q (CI)** |
| AX | mAPD | 1.17  (1.11 , 1.29) | -0.65  (-1.23 , -0.4) | 1.11  (0.96 , 1.36) | -0.49  (-1.63 , 0.2) |
| COR | r-lvAW | 1.3  (1.18 , 1.38) | -1.95  (-2.5 , -1.09) | 1.01  (0.96 , 1.09) | 0.02  (-0.46 , 0.33) |
|  | l-lvAW | 1.16  (1.04 , 1.26) | -1.01  (-1.67 , -0.39) | 1.09  (1.01 , 1.14) | -0.41  (-0.77 , 0.08) |
|  | cLLD | 0.97  (0.9 , 1.1) | 0.22  (-2.23 , 1.73) | 1.25  (1.14 , 1.31) | -5.34  (-6.53 , -3.25) |
|  | pcfLLD | 1.01  (0.93 , 1.08) | -0.5  (-2.44 , 1.61) | 1.08  (1 , 1.19) | -2.28  (-5.96 , 0.004) |
|  | cBPD | 1.19  (1.05 , 1.32) | -6.68  (-12.02 , -1.38) | 0.77  (0.72 , 0.91) | 8.18  (2.93 , 10.32) |
|  | tBPD | 1.18  (1.05 , 1.26) | -7.27  (-10.65 , -1.82) | 0.92  (0.81 , 1.01) | 3.85  (-0.25 , 8.61) |
| SAG | cFOD | 0.97  (0.89 , 1.04) | 1.08  (-2.8 , 5.54) | 1.11  (0.99 , 1.22) | -6.13  (-11.99 , 0.27) |
|  | tFOD | 0.83  (0.74 , 0.88) | 9.73  (7.11 , 14.11) | 1.08  (0.97 , 1.31) | -4.69  (-18.35 , 2.11) |
|  | ccL | 0.93  (0.88 , 1.02) | 1.04  (-0.41 , 1.95) | 1.01  (0.93 , 1.11) | -0.66  (-2.1 , 0.41) |
|  | pAPD | 0.95  (0.87 , 1.16) | 0.35  (-0.94 , 0.96) | 1.21  (1.08 , 1.42) | -1.31  (-2.69 , -0.6) |
|  | pCCD | 1.12  (0.97 , 1.26) | -0.81  (-1.64 , 0.16) | 1.21  (1.12 , 1.33) | -1.19  (-1.94 , -0.63) |
|  | vAPD | 0.87  (0.78 , 0.99) | 0.7  (0.1 , 1.17) | 1.01  (0.91 , 1.12) | -0.03  (-0.63 , 0.6) |
|  | vCCD | 1.35  (1.18 , 1.55) | -2.48  (-3.88 , -1.16) | 1.07  (0.94 , 1.23) | -0.73  (-2.07 , 0.07) |
|  | csA | 1.03  (0.9 , 1.14) | -0.77  (-9.56 , 7.8) | 1.37  (1.05 , 1.65) | -25.45  (-45.26 , -3.41) |

**Supplemental Table S2** NiftyMIC and MIALSRTK comparison in terms of 2D and brain reconstruction biometric measurements estimation differences. The estimation differences, expressed in millimeters (mm) or degrees (°), for each biometric measurement are reported as the average and 95 % Confidence Interval (CI) ranges of all measurement differences.

| **Orthogonal**  **Orientation** | **Biometric Measure** | **NiftyMIC** | **MIALSRTK** |
| --- | --- | --- | --- |
|  |  | **Mean differences (CI)** | **Mean differences (CI)** |
| AX | mAPD (mm) | -0.02 (-0.43 , 0.38) | 0 (-0.48 , 0.48) |
| COR | r-lvAW (mm) | 0.04 (-0.87 , 0.96) | -0.04 (-0.65 , 0.57) |
|  | l-lvAW (mm) | 0.06 (-0.82 , 0.94) | -0.08 (-0.89 , 0.74) |
|  | cLLD (mm) | 0.3 (-0.81 , 1.42) | 0.65 (-0.52 , 1.82) |
|  | pcfLLD (mm) | 0.38 (-1.75 , 2.51) | 0.43 (-1.75 , 2.6) |
|  | cBPD (mm) | -0.14 (-3.08 , 2.79) | 0.4 (-2.53 , 3.32) |
|  | tBPD (mm) | -0.4 (-3.4 , 2.6) | 0.05 (-3.38 , 3.49) |
| SAG | cFOD (mm) | 0.58 (-2.65 , 3.82) | 0.76 (-3.52 , 5.04) |
|  | tFOD (mm) | -0.18 (-3.73 , 3.36) | 0.06 (-4.1 , 4.22) |
|  | ccL (mm) | -0.13 (-1.78 , 1.52) | 0.39 (-1.37 , 2.16) |
|  | pAPD (mm) | -0.02 (-0.69 , 0.64) | 0.04 (-0.73 , 0.8) |
|  | pCCD (mm) | 0.06 (-0.5 , 0.63) | 0.01 (-0.53 , 0.56) |
|  | vAPD (mm) | 0.03 (-0.46 , 0.52) | -0.03 (-0.64 , 0.59) |
|  | vCCD (mm) | -0.09 (-1.16 , 0.97) | 0.27 (-0.86 , 1.4) |
|  | csA (°) | -0.03 (-8.07 , 8.01) | -0.47 (-10.37 , 9.44) |

**Supplemental Table S3** NiftyMIC and MIALSRTK comparison in terms of slope and intercept values, derived from the biometric measurements performed on TSE sequences. The slope (m) and intercept (q) values, reported with the 95% Confidence Interval (CI), are estimated using the Passing-Bablok regression analysis on each biometric measurement.

| **Orthogonal**  **Orientation** | **Biometric Measure** | **NiftyMIC** | | **MIALSRTK** | |
| --- | --- | --- | --- | --- | --- |
|  |  | **m_TSE_ (CI)** | **q _TSE_ (CI)** | **m _TSE_ (CI)** | **q _TSE_ (CI)** |
| AX | mAPD | 0.95  (0.8 , 2.11) | 0.39  (-5.18 , 1.13) | 1.15  (0.71 , 2) | -0.79  (-4.44 , 1.14) |
| COR | r-lvAW | 1.21  (1.14 , 1.34) | -1.3  (-2.17 , -0.83) | 1.22  (1.12 , 1.28) | -1.18  (-1.69 , -0.5) |
|  | l-lvAW | 1.6  (1.33 , 1.77) | -3.92  (-5.06 , -2.14) | 1.12  (1.08 , 1.29) | -0.58  (-1.75 , -0.38) |
|  | cLLD | 0.91  (0.79 , 1.17) | 1.53  (-3.82 , 4.12) | 1.14  (1 , 1.27) | -3.44  (-5.83 , -0.49) |
|  | pcfLLD | 1.07  (0.95 , 1.17) | -1.93  (-5.12 , 1.24) | 1.3  (1.19 , 1.49) | -8.94  (-14.13 , -5.95) |
|  | cBPD | 1.13  (0.95 , 1.29) | -5.14  (-11.24 , 1.97) | 0.61  (0.55 , 0.74) | 13.95  (9.6 , 16.59) |
|  | tBPD | 1.09  (1.03 , 1.46) | -4.4  (-19.5 , -1.67) | 0.84  (0.68 , 0.97) | 7.23  (0.71 , 13.39) |
| SAG | cFOD | 1  (0.83 , 1.14) | -0.29  (-6.57 , 8.24) | 1.43  (1.11 , 1.8) | -22.36  (-42.65 , -6.04) |
|  | tFOD | 0.85  (0.72 , 1.02) | 7.93  (-0.8 , 15.75) | 1.39  (1.33 , 1.83) | -22.92  (-48.57 , -18.96) |
|  | ccL | 0.78  (0.76 , 0.88) | 3.71  (2.23 , 4.04) | 1.31  (1.26 , 1.45) | -4.64  (-6.62 , -3.87) |
|  | pAPD | 0.96  (0.83 , 1.33) | 0.43  (-1.78 , 1.22) | 1.46  (0.49 , 1.81) | -2.74  (-5.22 , 3.69) |
|  | pCCD | 1.17  (0.91 , 1.6) | -1.03  (-3.59 , 0.72) | 1.91  (-1.43 , 2.1) | -5.49  (-6.27 , 14.45) |
|  | vAPD | 0.86  (0.61 , 0.998) | 0.73  (0.05 , 1.86) | 0.94  (0.67 , 1.01) | 0.23  (-0.15 , 1.65) |
|  | vCCD | 1.16  (1.13 , 1.18) | -0.82  (-1.05 , -0.53) | 1  (0.87 , 1.3) | -0.53  (-2.55 , 0.44) |
|  | csA | 0.98  (0.81 , 1.08) | 2.17  (-5.17 , 13.4) | 1.17  (0.85 , 1.56) | -9.27  (-32.49 , 13.75) |

**Supplemental Table S4** NiftyMIC and MIALSRTK comparison in terms of slope and intercept values, derived from the biometric measurements performed on b-FFE sequences. The slope (m) and intercept (q) values, reported with the 95% Confidence Interval (CI), are estimated using the Passing-Bablok regression analysis on each biometric measurement.

| **Orthogonal**  **Orientation** | **Biometric Measure** | **NiftyMIC** | | **MIALSRTK** | |
| --- | --- | --- | --- | --- | --- |
|  |  | **M_b-FFE_ (CI)** | **q_b-FFE_ (CI)** | **m_b-FFE_ (CI)** | **q_b-FFE_ (CI)** |
| AX | mAPD | 1.22  (1.08 , 1.48) | -0.91  (-2.02 , -0.28) | 1.04  (0.8 , 1.36) | -0.04  (-1.95 , 1.04) |
| COR | r-lvAW | 1.17  (0.998 , 1.36) | -1.38  (-2.77 , -0.22) | 0.89  (0.86 , 0.93) | 0.75  (0.51 , 0.82) |
|  | l-lvAW | 0.93  (0.87 , 1.02) | 0.26  (-0.15 , 0.68) | 1.01  (0.92 , 1.09) | 0.08  (-0.53 , 0.65) |
|  | cLLD | 1.1  (0.95 , 1.26) | -2.24  (-5.29 , 0.67) | 1.27  (1.13 , 1.45) | -5.86  (-9.64 , -2.75) |
|  | pcfLLD | 0.94  (0.8 , 1.13) | 1.32  (-3.89 , 4.96) | 0.92  (0.84 , 1.002) | 2.12  (0.43 , 4.23) |
|  | cBPD | 1.32  (1.06 , 1.51) | -11.43  (-17.62 , -1.99) | 1.07  (0.85 , 1.31) | -2.76  (-11.4 , 5.82) |
|  | tBPD | 1.2  (1.16 , 1.26) | -7.83  (-10.59 , -6.13) | 0.95  (0.8 , 1.31) | 3  (-13.1 , 9.58) |
| SAG | cFOD | 1.13  (0.97 , 1.37) | -7.07  (-18.63 , 1.23) | 0.99  (0.71 , 1.12) | 0.52  (-6.72 , 13.74) |
|  | tFOD | 0.81  (0.62 , 0.96) | 11.47  (3.77 , 21.3) | 0.89  (0.8 , 1.01) | 6.65  (-0.18 , 11.61) |
|  | ccL | 1.07  (1.05 , 1.27) | -1.06  (-4.24 , -0.62) | 0.85  (0.79 , 0.98) | 1.67  (-0.24 , 2.78) |
|  | pAPD | 0.84  (0.69 , 0.95) | 1.1  (0.23 , 1.98) | 0.98  (0.96 , 1.15) | 0.04  (-1.06 , 0.37) |
|  | pCCD | 1.1  (0.96 , 1.25) | -0.75  (-1.65 , -0.003) | 1.16  (1.06 , 1.2) | -1.07  (-1.44 , -0.51) |
|  | vAPD | 0.97  (0.73 , 1.16) | 0.1  (-0.97 , 1.41) | 1.11  (0.75 , 1.12) | -0.43  (-0.52 , 1.43) |
|  | vCCD | 1.53  (1.23 , 1.73) | -3.95  (-5.39 , -1.74) | 1.12  (0.86 , 1.38) | -1.01  (-2.89 , 0.72) |
|  | csA | 1.21  (1.05 , 1.44) | -14.37  (-31.79 , -3.54) | 1.65  (1.15 , 1.79) | -46.81  (-56.85 , -10.95) |
